# CD49b Targeting Inhibits Tumor Growth and Boosts Anti-tumor Immunity

**DOI:** 10.3389/fonc.2022.928498

**Published:** 2022-07-04

**Authors:** Pamina Contreras-Kallens, Felipe Gálvez-Jirón, Javiera De Solminihac, Ahmed Elhusseiny, Wilfredo A. González-Arriagada, Francisca Alcayaga-Miranda, Randolph J. Noelle, Karina Pino-Lagos

**Affiliations:** ^1^ Centro de Investigación e Innovación Biomédica, Facultad de Medicina, Universidad de los Andes, Santiago, Chile; ^2^ Centro de Investigación e Innovación Biomédica, Facultad de Odontología, Universidad de los Andes, Santiago, Chile; ^3^ Cells for Cells, Santiago, Chile; ^4^ Consorcio Regenero, Chilean Consortium of Regenerative Medicine, Santiago, Chile; ^5^ IMPACT, Center of Interventional Medicine for Precision and Advanced Cellular Therapy, Santiago, Chile; ^6^ Dartmouth Medical School, Dartmouth College, Hanover, NH, United States

**Keywords:** Foxp3+ Treg, Tr1 cells, CD49b, tumor immunology, tolerance

## Abstract

The suppressive function of T-regulatory cells (Tregs) can have a detrimental effect on immune responses against tumor cells. Within the Treg cells subset, a new non-classical population has been reported, which expresses high levels of CD49b molecule and, depending on their activation status, can also express the canonical Tregs transcription factor Foxp3. In this report, we sought to characterize Tregs subsets in a murine melanoma model and disrupt the CD49b/CD29 axis by administering an anti-CD29 antibody in tumor-bearing mice. Our data shows that whereas in the draining lymph nodes, the Tr1 cells subset composes <5% of CD4+ T cells, in the tumor, they reach ∼30% of CD4+ T cells. Furthermore, Tr1 cells share the expression of suppressive molecules, such as Nrp-1, PD-1, and CD73, which are highly expressed on Tr1 cells found in tumor-infiltrating leukocytes (TILs). Regardless of the phenotypic similarities with cTreg cells, Tr1 cells display a low proliferative activity, as shown in the kinetics and the incorporation of 5-bromodeoxyuridine (BrdU) experiments. With the intent to impact on Tr1 cells, we administered anti-CD29 antibody into tumor mice, observing that the treatment effectively inhibits tumor growth. This effect is at least mediated by the enrichment of pro-inflammatory T cells, including IFN-γ+ cTreg and IFN-γ+ Tr1 cells (with reduced expression of IL-10), plus Th1 and Tc cells. In this study, we present Tr1 cell characterization in tumor-bearing animals and introduce CD29 as a target for tumor therapy, supported by a meta-analysis indicating that CD29 is present in human biopsies.

## Introduction

T-regulatory (Treg) cells are essential for modulating immune cell activity and maintaining immunological tolerance. However, their suppressive function can have detrimental effects on immune responses against tumor cells (1). Thus, it is of great importance to study factors affecting their inhibition efficiency for the improvement of anti-tumor therapies. To date, several subsets of Treg cells have been described, which can be divided into two major groups, namely, Foxp3+ Treg cells and Foxp3− Treg cells, depending on the expression of the transcription factor Foxp3 (2). Although the population of Foxp3+ Treg cells can be further subdivided into thymus- or periphery-generated Foxp3+ Treg cells, in this report, we will refer to them collectively as conventional Treg (cTreg) cells based on the presence of Foxp3+ and not them by their origin. Foxp3− Treg cells were first described by Bachetta et al. who isolated CD4+ T cells with high levels of interleukin (IL)-10 and low levels of IL-2 production from a severe combined immunodeficiency (SCID) patient who had successfully been treated with multiple HLA-mismatched bone marrow transplants (3). Then, it was subsequently shown that these cells had an immunomodulatory effect *in vivo* and were generated after the chronic activation of CD4+ T cells in the presence of IL-10 and were named Tr1 cells (4). These cells were also shown to be distinct from cTreg cells, as they lacked the expression of the Foxp3 marker but possessed a strong immunosuppressive effect, mediated mainly through the secretion of IL-10 (5). Other immunomodulatory mechanisms have been reported for Tr1 cells since they were first described, such as the secretion of granzyme B, perforin, and transforming growth factor β (TGF-β), which has been shown to inhibit T-cell responses (6).

The role of Tr1 cells in immune modulation has been demonstrated in several pre-clinical disease models, such as experimental autoimmune encephalomyelitis, inflammatory bowel disease, allogeneic pancreatic islet transplantation, and collagen-induced arthritis, which supports their biological relevance to control immune reactions (reviewed in Roncarolo et al., 2018). The lack of a specific marker for the identification of Tr1 cells impeded research on their clinical application, as they were identified by their specific cytokine secretion pattern, which includes the secretion of IL-5, transforming growth factor β (TGF-β), interferon-γ (IFN-γ), and absence of IL-15 and IL-2 (5). In 2013, Gagliani et al. reported that Tr1 cells could be identified by their co-expression of CD49b and lymphocyte activation gene 3 (LAG-3) on their surface, thus facilitating further research into their clinical application (Gagliani et al., 2013). CD49b is an integrin α chain that associates with the β1 integrin CD29, forming the CD49b/CD29 complex, while LAG-3 is a co-inhibitory receptor expressed by T cells (7, 8). The discovery of these two markers led to the investigation of the presence and expansion of Tr1 cells, identified by the co-expression of these markers in conditions such as chronic graft-versus-host disease and liver tumors in humans (9, 10). However, while the role of Treg in the tumor microenvironment (TME) has been widely described, the mechanisms by which Tr1 cells mediate their immunomodulatory effects and how their presence contributes to the heterogeneity of Tregs in the TME remains to be elucidated.

The identification of Tr1 cells by the combination of these two markers has been questioned, as it was reported that, in a EAE pre-clinical model, while half of antigen-specific CD4+IL-10+ were positive for CD49b, LAG-3 was expressed in the majority of CD4+ T cells, regardless of their IL-10 production (11). Similar results were found *in vitro*, in which only a small proportion of CD4+ IL-10+ cells were positive for both markers, while more than half of this subset was CD49b+ (11). The use of CD49b alone as a Tr1 cell marker has been used in collagen-induced arthritis (CIA) mice model, to identify potent Treg cells that recirculate through peripheral tissues and skin in mice and also as a prognostic biomarker of Tr1 cells in patients receiving antiviral therapy for recurrent hepatitis C (12–14). We therefore decided to characterize and phenotype Tr1 cells, using CD49b as a marker, in tumor-bearing animals, emphasizing the relevance of the CD49b/CD29 axis in anti-tumor responses.

In this study, we found that CD49b+Foxp3−CD4+ Tr1 cells accumulate at the tumor site at higher frequencies than cTregs from early in the tumor development. In addition, Tr1 cells express Neuropilin-1 (Nrp-1), programmed cell death protein 1 (PD-1), and CD73, at an elevated level when they reside at the tumor site compared to tumor-draining lymph nodes (TdLNs). In contrast to cTreg cells, Tr1 cells show very low proliferative capacity in lymph nodes and tumors. Moreover, with the objective of interrupting Tr1 cells in tumors, we administered anti-CD29 antibody into tumor-bearing animals, which resulted in a dramatic impairment of tumor growth accompanied by an increased presence of IFN-γ+ cTreg and IFN-γ+ Tr1 cells with reduced expression of IL-10, in addition to an increase in IFN-γ+CD4+T cells (Th1) and IFN-γ+CD8+T cells (cytotoxic T cells or Tc).

In summary, this report demonstrates that Tr1 cells, not only cTreg cells, are present at the tumor site in the early stages of the tumor development and that the disruption of the CD49b/CD29 axis prevents tumor growth by at least targeting CD4+T and CD8+T cells function. At the same time, we introduce CD29 as a novel target for tumor eradication.

## Methods

### Animals

Six- to eight-week-old wild-type C57BL/6 and Foxp3^GFP^ (C57BL/6 background) reporter and RAG-KO mice were used. All mice were maintained under pathogen-free conditions, with 12 h light/dark cycle and food water *ad libitum*. This study was carried out after the revision and approval of its experimental protocol in accordance with the recommendations of the bioethical Committee guidelines from the Faculty of Medicine, Universidad de los Andes and the Agencia Nacional de Investigación y Desarrollo (ANID).

### Cell Cultures

B16 melanoma, MB49, and EL-4 cell lines were donated by Dr. Daniela Sauma (from Facultad de Ciencias, Universidad de Chile). B16, MB49, and EL-4 cells were cultured in Roswell Park Memorial Institute (RPMI) 1640 medium (Sigma, Milano, Italy) supplemented with 10% fetal bovine serum (FBS) (Grand Island, New York, USA), HEPES (Gibco, Great Britain), 1% penicillin/streptomycin, and 50 μM β-mercaptoethanol, for 5 days at 37°C in a humidified atmosphere containing 5% CO_2_. When cells reach 70% confluence, trypsin treatment was used for collecting them (B16 and MB49), and trypan blue staining was performed for cell counting.

### Tumor Inoculation and Anti-CD29 Treatment

A total of 2 × 10^5^ B16 melanoma cells (or 1 × 10^5^ B16 cells for RAG-KO mice), 2.5 × 10^5^ MB49 cells, and 1.25 × 10^5^ EL-4 cells were administered *via* intra-dermal in 100 μl of sterile phosphate-buffered saline (PBS) 1× into the right flank of mice. Tumor size was measured every other day using a digital caliper. For anti-CD29 treatment, mice received 200 ng/mouse *via* intraperitoneal administration three times per week starting injections when tumors were palpable (around days 9–11 post-inoculation). As control, the same volume of PBS 1× was administered.

### Tumor-Infiltrating Leukocytes Isolation

Mice were euthanized between day 18 and 20 after tumor induction (avoiding a volume >3,000 mm^3^). Tumor-draining lymph nodes (TdLNs) and tumor mass were harvested for analysis. Cell suspensions were prepared, disrupting the tissue directly on cell strainers (40 µm, BD Falcon East Rutherford, New Jersey, USA). For tumor-infiltrating leukocytes (TILs) suspensions, the solid tumor was first mechanically disrupted, followed by enzymatic digestion for 40 min at 37°C using a DNase and Liberase solution in RPMI (50 and 250 μg/ml, respectively) (both enzymes from Sigma, Milano, Italy). Cell suspensions were then prepared using a 40%/70% Percoll (GE Healthcare, IL, USA) gradient centrifugation as previously described (15). In brief, after the enzymatic digestion, the cells were washed once in PBS, and the pellet was resuspended in 4 ml of 40% isotonic Percoll and overlaid on 3 ml of 70% isotonic Percoll. Cells were then centrifuged at 500*g* for 40 min, and TILs were collected from the 40% to 70% interface, washed once in PBS, and counted in a Neubauer Chamber.

### Harris Hematoxylin and Eosin Staining

To perform the processing of the histology sections, animals were euthanized, and a surgical excision was performed to extract the tumor mass including a border of healthy tissue. Samples were stored in paraformaldehyde (PFA) to fix. After a week, the tissue was placed in 70% alcohol and stored at 4°C. Next, the samples were dehydrated with a gradient of alcohols from 70% alcohol to xylol and then embedded in paraffin, which was done using the tissue transfer processor equipment (model: BW-12EP). Paraffin blocks were prepared in the embedding equipment (Tissue-Tec TEC). A microtome was used to obtain 5-µm-thick tissue sections, which were placed on a float bath at 45°C. The sections were then mounted on the slides and left to dry for subsequent tissue staining. Hydration of tissues was performed in an alcoholic gradient passing through xylol, absolute alcohol, 95% ethanol, 70% ethanol, 50% ethanol, and water. After this, the tissue was stained with Harris hematoxylin and alcoholic eosin, followed by a dehydration step performing the same alcohol gradient in the reverse direction.

### Flow Cytometry Analysis

Single-cell suspensions were stained with anti-CD45 (clone I3/2,3), anti-CD4 (clone RM4-5), anti-CD8 (clone 53-6.7), anti-CD49b (clone HMα2), anti-CD73 (clone TY/11.8), anti-PD-1 (clone 29F.1A12), anti-Nrp-1 (clone 3E12), anti-IL-10 (clone JES5-16e3), anti-Foxp3 (clone FJK-165), anti-granzyme B (clone QA16A02), and anti-IFN-γ clone XMG1.2) coupled to FITC, PE, PE-Cy7, PerCP, PerCP-Cy7, and APC (all from Biolegend, NJ, USA). For intracellular cytokine staining, cells were stimulated for 5 h at 37°C adding PMA (50 ng/ml) (Sigma, Milano, Italy), ionomycin (1 mg/ml) (Sigma, Milano, Italy), and Brefeldin A (10 mg/ml) (eBioscience, CA, USA). Then, cells were fixed using a permeabilization kit (Biolegend or BD, USA) according to the manufacturer’s procedure and subsequently stained for intracellular markers. Flow cytometric data were acquired using FACS CantoII (BD Immunocytometry System, San Jose, USA) and analyzed with FlowJo software (Tree Star, OH, USA).

### viSNE Visualization

To perform visualization of complex flow cytometry data, we used the Cytobank computational tool viSNE (visualization of t-Stochastic Neighbor Embedding), which generates a two-dimensional map in which cell distance represents distance between cell parameters in high-dimensional space (16). Thus, cells that are phenotypically similar for the analyzed markers will be closer in a viSNE map (Becher et al. 2014; Leelatian et al., 2015). To generate viSNE maps, samples were uploaded to Cytobank; live single cells were gated based on cell size and length and negative to Zombie Dye viability staining and later gated in the CD4+ subset. Then, between 150,000 and 160,000 cells were subsampled from the data. After subsampling, viSNE was run at default parameters (1,000 iterations, random seed, perplexity = 30, theta = 0.5). viSNE maps were visualized using the Cytobank interface, which was used to generate figures (color coding by marker expression levels).

### Assessment of Cell Proliferation by 5-Bromodeoxyuridine Labeling

To assess cTregs and Tr1 cells proliferation in TdLN and TILs, 200 μl of 5-bromodeoxyuridine (BrdU) solution was injected into mice (100 mg of BrdU per kilogram, in sterile PBS 1×) *via* intraperitoneal administration. Two BrdU pulses were used in the experimental procedure, the first one at 12 days after tumor inoculation and the second one at day 15. At day 20, mice were euthanized, TdLNs plus tumor mass were harvested, and cell suspensions were prepared. Bone marrow cells were used as positive control. All cell suspensions were stained for the surface markers CD45, CD4, and CD49b. Then, cells were fixed and permeabilized using a solution of 1% paraformaldehyde/0.01% Tween-20 as previously described (17). After overnight incubation, cells were treated with DNAse I (Sigma, Milano, Italy) solution for 10 min at room temperature (RT). Finally, cells were stained with anti-BrdU (clone 3D4) and anti-Foxp3 (Invitrogen, CA, USA) antibodies and later analyzed by flow cytometry.

### 7AAD/Annexin V Assay

B16, MB49, and EL4 cells were seeded in a six-well plate until reaching ~90% of confluency in supplemented RPMI. Cells were treated with 1 and 10 ng/ml of anti-CD29 for 4 h. Armenian hamster IgG (Biolegend, San Diego, California, USA) was used as control. After 4 h, cells were collected and washed twice with cold PBS and then resuspended in 1× binding buffer (Biolegend, USA) at 10^6^ cells/ml. One hundred microliters of the cell suspension was transferred onto a 5-ml culture tube. Five microliters of Annexin-v APC and 7-AAD was added per sample. The cells were gently vortexed and incubated for 20 min at room temperature. One hundred microliters of the binding buffer was added to each tube, and the samples were analyzed by fluorescence-activated cell sorting (FACS).

### Differential Gene Expression of CD29 on Human Cancer Cells

Publicly available RNA-Seq data from Bladder Cancer (BLCA), Skin Cutaneous Melanoma (SKCM), and Genomic Variation in Diffuse Large B-Cell Lymphomas (NCICCR-DLBCL) and Burkitt Lymphoma Genome Sequencing Project (CGCI-BLGSP) were downloaded directly from The National Cancer Institute GDC Data portal (https://portal.gdc.cancer.gov). These data included HTSeq-Counts of matched samples with reported clinical/pathological stages (**Table 1**). RNA-Seq data from healthy tissues (control) were downloaded from the Genotype–Tissue Expression (GTEx) (v8) project (https://gtexportal.org). Differential gene expression was assessed using R/edgeR by applying trimmed mean of M-values (TMM) normalization. Respective cancer and normal tissue sample data were normalized together to avoid composition bias. Log2-CPM transformation was utilized to obtain normalized expression values. The expression of CD29 (ENSG00000150093) was further evaluated.

**Table 1:** Number of human cancer samples for CD49b and CD29 gene expression analysis.

	Number of Samples
**Bladder**	
Control	21
Cancer	412
Stage I	4
Stage II	130
Stage III	142
Stage IV	136
**Melanoma**	
Control (skin cells, Sun Exposed (Lower leg))	701
Cancer	412
Stage I	77
Stage II	140
Stage III	171
Stage IV	24
**Lymphoma**	
Control (whole blood)	755
Cancer	420
Stage I	59
Stage II	121
Stage III	113
Stage IV	127

### Statistical Tests

Statistical significance was determined using the GraphPad Prism software version 8 (GraphPad Software, CA, USA), and depending on the data distribution, parametric, non-parametric, or ANOVA tests were used.

## Results

### Distribution of Conventional Treg and Tr1 Cells in Tumor-Bearing Mice

**Figure 1:**
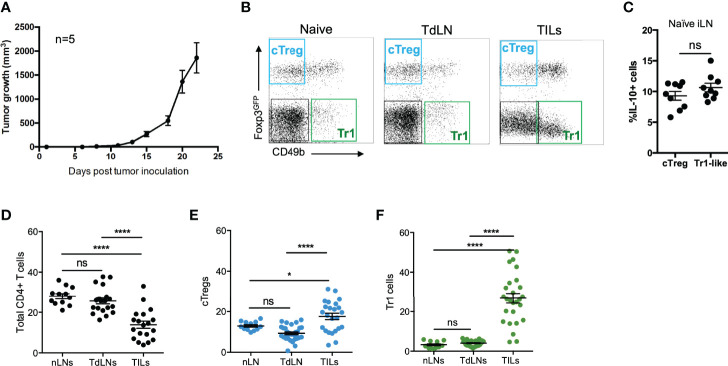
Tr1 cells are more abundant at the tumor site than conventional Tregs. **(A)** A total of 2 × 10^5^ B16 cells were injected into the right flank of C57BL6^GFP^ reporter mice, and tumor growth was monitored and measured every other day. Between day 18 and 20 post-inoculation, tumor-draining lymph nodes (TdLNs) and tumor-infiltrating lymphocytes (TILs) were collected for flow cytometry analysis. **(B)** Representative dot plots from naive inguinal lymph nodes (nLNs), tumor draining lymph nodes (TdLNs), and tumor-infiltrating leukocytes (TILs) depicting the gating strategy for the study of conventional Treg cells (cTreg) considered as CD4+Foxp3^GFP+^CD49b− T cells and Tr1 cells, considered as CD4+Foxp3^GFP−^CD49b+ T cells. The frequencies of IL-10+ cells in cTreg and Tr1 cells subsets **(C)**, of total CD4+ T cells **(D)**, cTregs **(E)**, and Tr1-like cells **(F)** from naive and tumor-bearing mice are shown in the corresponding graphs. * p < 0.05; **** p < 0.0001 according to one-way ANOVA (multiple comparisons) for panels **(D–F)** and Mann–Whitney test for panel **(C)**; ns, not significant. Mean ± SEM. n = 3 independent experiments with five to eight mice per group.

To better understand the dynamics of both cTreg and Tr1 cells, we used a widely accepted tumor model in which immunocompetent recipient animals are inoculated with syngeneic B16 melanoma cell line (18). To facilitate the identification of cTreg cells (Foxp3+), we used Foxp3^GFP^ reporter mice (19). Tumor draining lymph nodes (TdLN) and tumor-infiltrating lymphocytes (TILs) were harvested from tumor-bearing mice (or inguinal lymph nodes from control naive animals) at day 20 post-tumor injection, and the expression of CD45, CD4, Foxp3, CD49b, and IL-10 was analyzed by flow cytometry. In this study, we considered cTreg cells as CD4+ T cells expressing Foxp3^GFP^ but lacking CD49b, and Tr1 cells as CD4+CD49b+ T cells lacking Foxp3^GFP^ as previously described (13). In **Figure 1A**, we show the kinetics of tumor growth (B16 melanoma), and in **Figure 1B**, dot plots depicting the expression of Foxp3^GFP^ and CD49b on previously gated CD4+ T cells obtained from the indicated organs are shown, in which cTreg cells are highlighted in the blue gate and Tr1 cells in the green gate. To corroborate that this gating strategy is considering suppressive cells, we analyzed the production of IL-10 by these Treg subsets, finding that ∼10% of cTreg and Tr1 are IL-10+ in naive animals (**Figure 1C**).

Next, we studied the presence of CD4+ T-cell subsets in tumor-bearing animals. As depicted in **Figures 1D–F**, the frequencies of total CD4+ T cells remain similar when comparing lymph node cells from naive (nLN) and tumor mice (TdLN) (∼25%), while in the TILs, the frequency of total CD4+ T cells is considerably lower (∼10%). Not surprisingly, cTreg cells frequency is double (~20%) in TILs, compared to the frequency observed in the nLN and TdLN (**Figure 1E**). Strikingly, while ≤5% of CD4+ T cells in the nLN and TdLN were identified as Tr1 cells, they comprise almost 30% in TILs (**Figure 1F**).

### Tr1 Cells Frequency Increases Over Time in the Tumor Site, Differing in Proliferative Function to cTreg Cells

**Figure 2:**
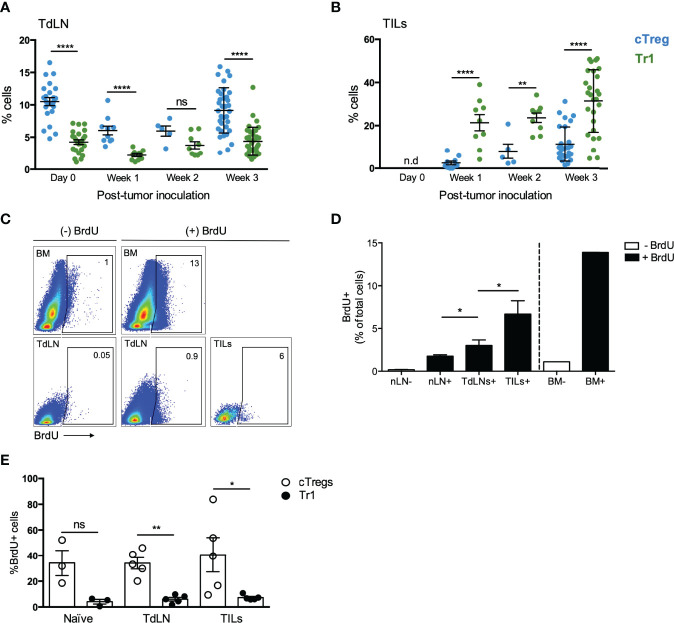
cTreg cells are highly proliferative in comparison to Tr1 cells *in vivo*. To evaluate the accumulation of cTreg and Tr1 cells, mice were treated as described in the legend of **Figure 1**. Mice were euthanized after 1-, 2-, or 3-week post-inoculation for analyzing the frequencies of cTreg and Tr1 cells in TdLN **(A)** and TILs **(B)**. In another set of experiments, tumor-bearing animals were used to study Treg cells proliferation through the injection of BrdU at days 12 and 15 after tumor induction (more details in *Materials and Method*). Organs were harvested at day 20, including bone marrow (BM) as positive control. Naive mice that had not received the BrdU injection were used as negative control. **(C)** Representative plot depicting BrdU+ cells from different samples. **(D)** Graph showing the frequencies of BrdU+ cells in different tissues: naive lymph nodes of mice not injected (nLN−, negative control) or injected with (nLN+), tumor-draining lymph nodes (TdLN+), and tumor-infiltrating lymphocytes (TILs+) of mice injected with BrdU, and BM of mice injected or not with BrdU (BM+, positive control; BM− negative control). **(E)** BrdU+ cells on cTreg and Tr1 cells populations in inguinal lymph nodes of naive animals, TdLNs, and within the tumor. All results are shown as mean ± SEM. n = 2–5. * p < 0.05; ** p< 0.01; ****p < 0.0001; ns, not significant according to Mann–Whitney test.

To elucidate the mechanism behind the high Tr1 cells frequency in the tumor site, we measured their frequency in the TILs fraction at different time points of tumor growth. Interestingly, while cTreg frequencies increase in the TILs fraction overtime after tumor inoculation from ~2% at week 1 to ~10% at week 2 and then ~15% at week 3, Tr1 cells frequency is high from early time points, comprising ~20% at week 1 and week 2 and ~30% at week 3 (**Figure 2B**). In contrast, cTreg levels are higher than Tr1 cells levels in TdLNs at almost all time points, except in week 2 (**Figure 2A**). Thus, high frequencies of the Tr1 cell subset are established early during tumor growth, while cTreg cells frequency builds up over time. This sheds light on the role of Tr1 cells as early players in inducing the immunosuppressive tumor microenvironment.

It has been well demonstrated that cTreg cells can infiltrate the tumor site and, due to the immune regulatory signals received within the tumor, differentiate from CD4+Foxp3^GFP-^ T cells to CD4+Foxp3^GFP+^ T cells and expand and accumulate at this location (20, 21). In contrast, the dynamics of Tr1 cells are less understood, and although they have been described to show low proliferative capacity, they can proliferate in the presence of IL-15 (5, 22, 23). Then, with the aim of better understanding on how high Tr1 frequencies are established within the tumor, we designed an experiment to evaluate the proliferative status of cTreg and Tr1 cells in tumor-bearing animals. For this purpose, mice were inoculated with B16 melanoma cells as mentioned before, and at days 12 and 15 post-tumor inoculation, a solution of bromodeoxyuridine (BrdU) (100 mg/kg) was administered *via* intraperitoneally (i.p.) to mice. At day 20, TdLNs and tumors were harvested and stained for flow cytometry analysis. Our results indicate that the tumor contains ∼7% of BrdU+ cells in comparison with ∼2.5% found in TdLN and ∼1% in naive control nLN (**Figures 2C, D**). Interestingly, while over ∼30% of BrdU+ cells corresponded to cTreg cells, only ∼5% corresponded to Tr1 cells (in all sites analyzed), thus suggesting that cTreg cells are more proliferative than Tr1 cells in the tumor microenvironment (**Figure 2E**) and that the high frequency of Tr1 cells found in the tumor is not due to the expansion of these cells at the selected time points.

### Tr1 Cells Expression of Immunosuppressive Markers

**Figure 3:**
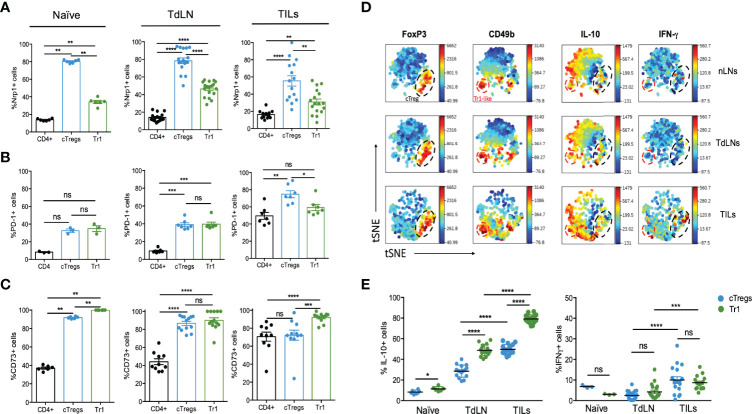
Tr1 cells express Nrp-1, PD-1, and CD73. A total of 2 × 10^5^ B16 cells were injected into the right flank of mice, and tumor growth was monitored and measured with caliper every other day. Between day 18 and 20 post-inoculation, mice were euthanized for cell phenotyping in naive inguinal lymph nodes (naive), tumor-draining lymph nodes (TdLNs), and tumor-infiltrating lymphocytes (TILs) using flow cytometry. The frequencies of CD4+ T cells, cTreg, and Tr1 cells expressing Nrp-1 **(A)**, PD-1 **(B)**, and CD73 **(C)** were evaluated as shown in the indicated graphs. Data from two independent experiments are shown as the mean ± SEM. n = 3–6 mice per experiment. * p < 0.05; ** p< 0.01; *** p < 0.001; ns, not significant according to one-way ANOVA (multiple comparison). **(D, E)**. Inguinal (naive) and tumor-draining lymph nodes (TdLNs), in addition to tumor-infiltrating lymphocytes (TILs), were processed for the analysis of CD4, Foxp3^GFP^, CD49b, IL-10, and IFN-γ expression by flow cytometry. **(D)** Representative viSNE map of concatenated files of all samples and concatenated individual populations. Frequencies of cTreg and Tr1 cells producing IL-10 (**E**, left) or IFN-γ (**E**, right) in the indicated cells/organs, obtained by intracellular staining. Data from three independent experiments are shown as the mean ± SEM. n = 4–5 mice per experiment. * p < 0.05; ** p< 0.01; **** p < 0.0001; ns, not significant according to one-way ANOVA (multiple comparisons).

In order to compare the regulatory phenotype between cTreg and Tr1 cells, we investigated the expression of other Treg-related molecules such as Nrp-1, PD-1, and CD73 (**Figure 3**). In naive animals, we found high frequencies of cTreg cells expressing Nrp-1 (∼80%) in comparison with Tr1 cells (∼40%) (**Figure 3A**). This difference followed the same trend in TdLN and TILs, in which the % of Nrp-1+cTreg cells was about twofold the frequencies of Tr1 cells (**Figure 3A**, top row). Regarding PD-1, the frequencies of PD1+ cTreg cells and Tr1 are similar in naive and TdLN (∼30% and 40%, respectively), whereas in TILs, the frequencies reach ∼80% of PD-1+ cTreg cells and ∼60% of PD-1+ Tr1 (**Figure 3B**, middle row). Lastly, we included CD73 in the analysis since this molecule plays an important role in Treg cells’ function and in tumor progression (24, 25), observing that the abundance of CD73+ cTreg and Tr1 cells are equivalent in naive and TdLN, ∼90% and ∼80%, respectively (**Figure 3C**, bottom panel), but in TILs, the frequencies of Tr1 cells (∼90%) are higher than cTreg cells (∼70%).

These data indicate that cTreg and Tr1 cells are differentially present and depicting different phenotype when residing in TdLN versus TILs. This difference is supported by the mean fluorescence intensity (MFI) of each marker, finding higher expression of Nrp-1 on Treg cells (versus Tr1 cells) in naive, TdLN, and TILs (**Supplementary Figure 1**, top row). As in the frequencies (or abundance) analysis, no differences in PD-1 expression by cTreg or Tr1 cells were observed in naive and TdLN, but in TILs, PD-1 expression in Tr1 cells is reduced threefold (**Supplementary Figure 1**, middle row). Lastly, CD73 is preferentially expressed by cTreg cells versus Tr1, in naive and TdLN, but in TILs, the expression of CD73 is higher in Tr1 cells (**Supplementary Figure 1**, bottom row). Overall, these data suggest that cTreg and Tr1 cells correspond to two different subsets of Treg cells and that the microenvironment (lymph node versus tumor) or location where the cells reside may impact their molecular signature.

To further complement a Treg cell signature, we included in our study other characteristic markers of Treg cells, using visualization of t-Stochastic Neighbor Embedding (viSNE) analysis, which generates a two-dimensional map in which cell distance represents the distance between cell parameters in high-dimensional space (16). This type of analysis allows a more objective and unbiased visualization of the cell population and the markers (26, 27). Thus, using viSNE, we corroborated that cTreg cells (Foxp3+) and Tr1 cells (Foxp3−CD49b+) conform to two separate and distinct populations inside the CD4+ T cell subset present in the lymph nodes of naive and tumor mice (nLN, TdLN) and in the TILs fraction, which can be identified by their Foxp3^GFP^ and CD49b expression (**Figure 3D**). We also observed that IL-10 was mainly secreted by these two populations in TdLNs and TILs, whereas IFN-γ+ cells were present in cTreg and Tr1 cells subsets in TILs (**Figure 3D**).

In terms of cytokine production levels, a higher frequency was observed in IL-10+ Tr1 cells than cTreg cells both in TdLN and in the TILs fraction (**Figure 3E**). In addition, IFN-γ production was not statistically different between the two subsets in any organ; however, it was observed that both subsets increased their production in TdLN compared to nLN, and in TILs compared to the TdLN (**Figure 3E**).

### Blockade of CD49b-CD29 Interaction Inhibits Tumor Growth

**Figure 4:**
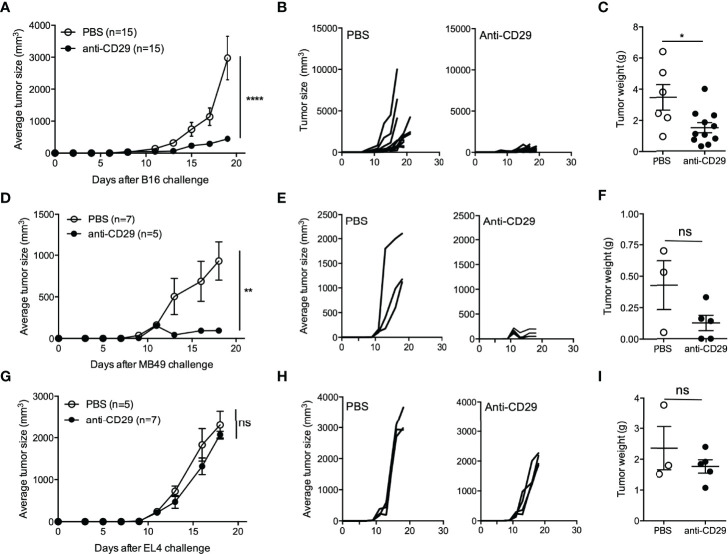
The blockade of CD49b/CD29 axis inhibits tumor growth. A total of 2 × 10^5^ B16, MB49, or EL-4 cells were injected into the right flank of C57BL6^GFP^ reporter mice. One hundred microliters of PBS 1× containing 200 ng of anti-CD29 antibody, or PBS alone as control, were administered to tumor-bearing animals when the tumors became palpable (approximately day 9 post-tumor inoculation). The treatment was given three times per week. At this time point, animals were euthanized for the isolation of TdLNs and tumors. Panels **(A–C)** depict tumor growth curves, in controls and treated animals, and tumor weight, respectively. The same analysis was carried out for animals inoculated with MB49 cells **(D–F)** and EL-4 cells **(G–I)**. For panels **(A–C)**, plots show data of two to three experiments with n = 5–6 animals per group. For panels **(D–F)** and **(G–I)**, one to two experiments were performed, with n = 3–7 animals per group. For panels **(A, D, G)**, Mann–Whitney test was applied at the last time point, and for panels **(C, F, I)**, one-way ANOVA was used. Data are shown as the mean ± SEM. * p < 0.05; ** p < 0.01; *** p < 0.001; **** p < 0.0001; ns, not significant.

Based on our results, we can summarize that draining lymph nodes and tumor site contain Treg cells populations at different proportions depending on the time point after tumor induction with different expressions of inhibitory molecules and distinct proliferative capacity. Considering the high proportion of Tr1 cells (considered as CD4+CD49b+Foxp3− T cells) found in the tumor site, we decided to test whether targeting CD49b could prevent tumor growth. Considering that CD49b (which is the α2 subunit of the integrin α2β1) is widely expressed on platelets and natural killer (NK) cells, we decided to target the β1 subunit (CD29) instead (28). The interaction between CD49b and CD29 forms the integrin α2β1, which acts as a receptor for collagen and mediates T-cell adhesion and migration to the inflammatory site (29). Furthermore, CD29 is related to costimulatory signaling in T cells, promoting their survival and cytokine production (30). As described above, Foxp3^GFP^ animals were inoculated with B16 melanoma cells, and around day 9 (when the tumor mass is palpable), a group of mice received anti-CD29 *via* i.p. injection (or PBS 1× as vehicle) three times per week. As shown in **Figures 4A, B**, the treatment with anti-CD29 antibody inhibits tumor growth and tumor weight (**Figure 4C**). Because of the impressive effect of the treatment, we repeated the experiment using other tumor cell lines injected subcutaneously. The tumor growth induced by subcutaneously injected MB49 cells, a syngeneic urothelial carcinoma cell line, was also dramatically blocked by the administration of anti-CD29, as previously observed in B16 melanoma-treated animals (**Figures 4D–F**). However, in animals injected with EL-4, a syngeneic lymphoma cell line, tumor growth was not affected (**Figures 4G–I**). Because CD29 interacts with CD49b, and the impact on tumor growth was not equal in the tumors studied, we sought that CD49b and cells may be relevant for the effectiveness of anti-CD29 treatment. As shown in **Supplementary Figure 2**, ∼40% of B16 cells and ∼80% of MB49 cells express CD49b; however, we did not detect CD49b expression on EL-4 cells. Similarly, we observed that ∼90% of both B16 and MB49 cells express CD29, whereas <1% of EL-4 cells are CD29+. These data show the relationship between the presence of these molecules on tumor cells and the outcome on tumor growth after anti-CD29 treatment.

### Anti-CD29 Treatment Skews T-Cell Signature Toward Pro-inflammation

Novel treatments to eliminate tumors are designed to stimulate immune effector cells while diminishing the effect of immunosuppressive mechanisms (31, 32). Anti-CTLA-4 or anti-PD-L1/PD-1 fully humanized antibodies, which target immune checkpoint molecules expressed by cTreg cells upon activation, have shown promising results in clinical studies treating various cancer types (31, 33). Although anti-CTLA-4 therapy was initially used to augment the activity of tumor-infiltrating CD8+ T and CD4+ T cells, it has been suggested that this therapy exerts its effects through a cTreg-depleting effect in the tumor site (34).

**Figure 5:**
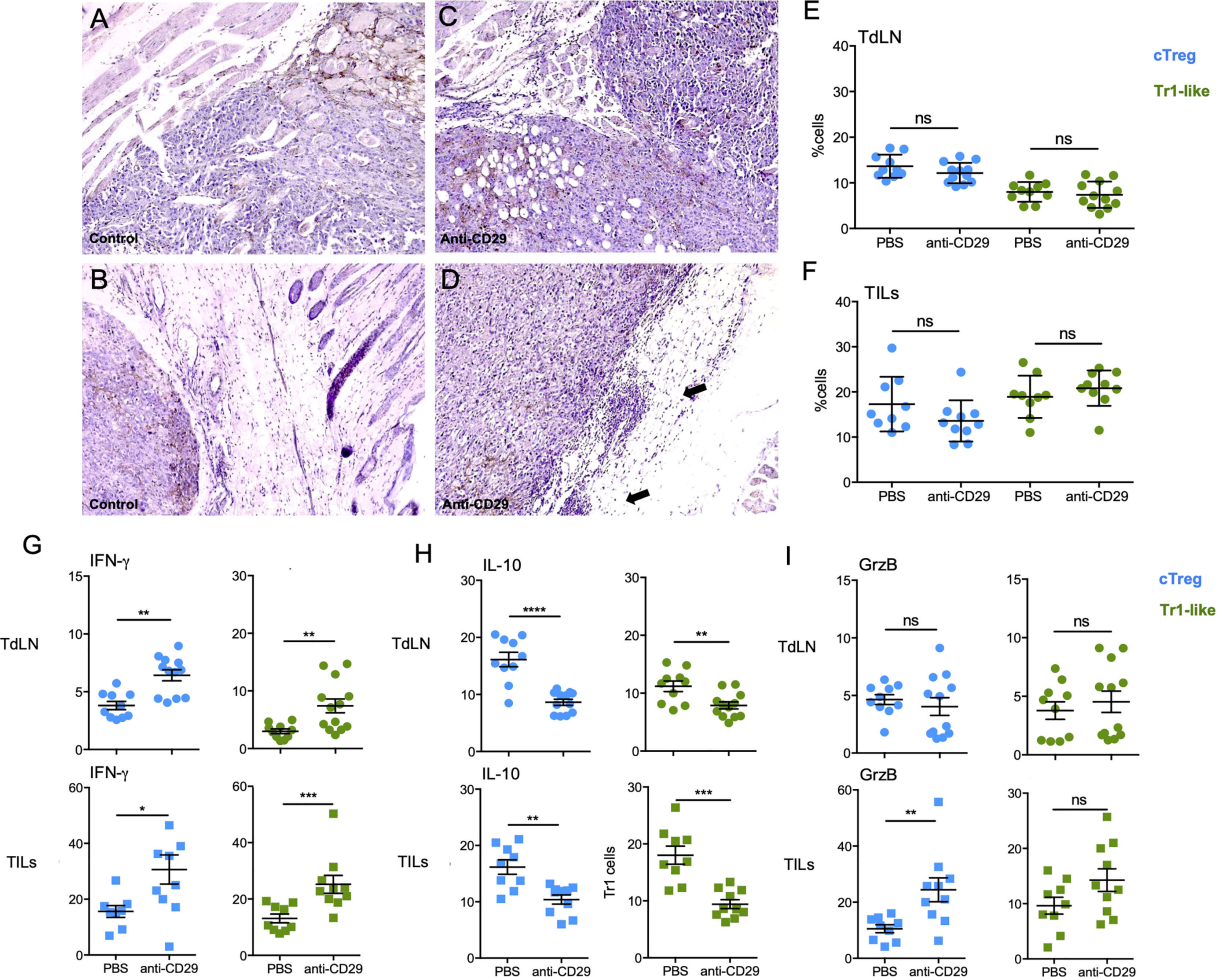
Administration of anti-CD29 antibody changes cTreg and Tr1 cells phenotype favoring a pro-inflammatory signature. A total of 2 × 10^5^ B16 were injected into the right flank of C57BL6^GFP^ reporter mice. One hundred microliters of PBS 1× containing 200 ng of anti-CD29 antibody, or PBS alone as control, was administered to tumor-bearing animals when the tumors became palpable (approximately day 9 post-tumor inoculation). The treatment was given three times per week. At this time point, animals were euthanized for the isolation of TdLNs and tumors. Tumors were processed for H&E staining, and cell suspensions were processed for antibody staining using anti-CD4, anti-CD49b, anti-IFN-γ, anti-IL-10, and anti-granzyme B (GrzB). Panels **(A, B)** correspond to tumors from control mice and panels **(C, D)** to tumors from anti-CD29 treated animals at 100×. **(E, F)** Graphs depicting the frequencies of cTreg and Tr1 cells in tumor-draining lymph nodes (TdLNs) and **(F)** tumor-infiltrating lymphocytes (TILs). Panels **(G–I)** show the frequencies of both cell populations expressing IFN-γ **(E)**, IL-10 **(F)**, or GrzB **(G)** in the indicated tissues and conditions. Data from two independent experiments are shown as the mean ± SEM. n = 5–6 mice per experiment. * p < 0.05; ** p< 0.01; *** p < 0.001; ns, not significant according to Mann–Whitney test.

Thus, based on the striking results using the treatment with anti-CD29 antibody, we decided to analyze tissue/tumor integrity by histology, in addition to several T-cell populations in TdLN and TILs, to determine the mechanism by which the antibody treatment is working. Hematoxylin and eosin (H&E) analysis of tumors harvested from mice treated with anti-CD29 showed a macroscopic reduction and a well-defined and less invasive tumor when observed under low magnification (40×) compared to control animals, which showed highly invasive tumors with neoplastic infiltration of muscle bundles and subcutaneous adipose tissue (data non shown). Under a higher magnification (100×), control tumors evidenced a more exuberant pleomorphism and involvement of adipocytes (**Figures 5A, B**), whereas in the tumors isolated from anti-CD29-treated animals, there is a clear delimitation of the tumor by the adipose tissue with some focal of lymphocytic infiltrate, evidencing a less aggressive behavior (**Figures 5C, D**). Next, we investigated cTreg and Tr1 cells; as shown in **Figures 5E, F**, the frequencies of both cell subsets cells were not changed upon anti-CD29 treatment, either in TdLN or TILs. Because these cell types remained unaltered, we studied their phenotype, finding that cTreg cells expressing IFN-γ were increased after anti-CD29 administration in TdLN (from ∼4% in PBS controls to ∼7% in anti-CD29 condition) and in TILs (from ∼15% in PBS controls to ∼30% in anti-CD29 condition) (**Figure 5G**, left column). The same trend was found for IFN-γ+ Tr1 cells in TdLN (from ∼2% in PBS controls and ∼8% in anti-CD29 condition) and in TILs (from ∼15% in PBS controls and ∼25% in anti-CD29-treated animals) (**Figure 5G**, graphs on the right). We also considered to examine the expression of IL-10, as this protein is pivotal for Treg-mediated immune tolerance and for promoting tumor growth; we measured the frequency of IL-10 producing Tregs by flow cytometry (35). We observed that in TdLN, both cTreg and Tr1 cells reduced the expression of IL-10: cTreg cells from ∼15% in PBS controls to ∼8% in anti-CD29-treated animals, and Tr1 cells from ∼10% in PBS controls to ∼8% in anti-CD29-treated animals (**Figure 5H**, top plots). In TILs, we obtained similar results where cTreg cells decreased IL-10 expression from ∼15% in PBS controls to ∼10% in anti-CD29-treated animals, and Tr1 cells from ∼18% in PBS controls to ∼9% in anti-CD29-treated animals (**Figure 5H**, bottom plots). Lastly, we checked the expression of granzyme B (GrzB), an important protein for the cytotoxic function of cells (36). cTreg and Tr1 cells residing in TdLN did not change GrzB expression after anti-CD29 administration (∼5% for both populations in both conditions) (**Figure 5I**, top plots). On the contrary, cTreg and Tr1 cells increased GrzB expression in TILs after anti-CD29 treatment, reaching statistical significance for GrzB+ cTreg cells only (from ∼10% in PBS control to ∼25% in anti-CD29 condition) (**Figure 5I**, bottom plots).

**Figure 6:**
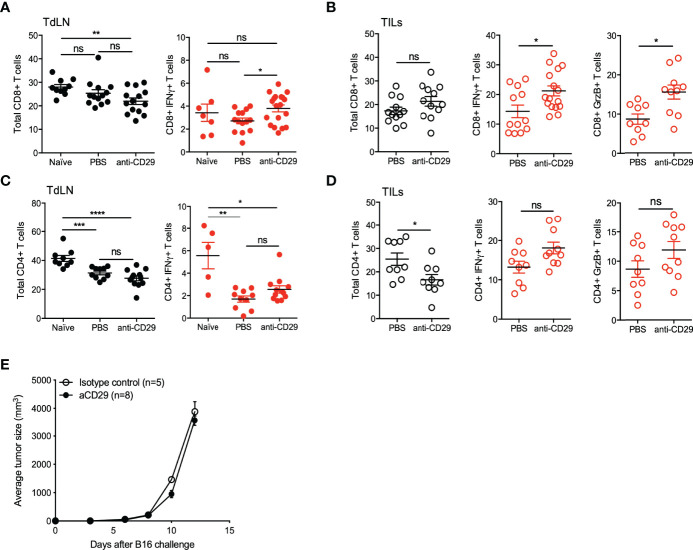
Disruption of CD49b/CD29 axis triggers effector CD4+ T and CD8+ T cells function. A total of 2 × 10^5^ B16 were injected into the right flank of C57BL6^GFP^ reporter mice. One hundred microliters of PBS 1× containing 200 ng of anti-CD29 antibody, or PBS alone as control, were administered to tumor-bearing animals when the tumors became palpable (approximately day 9 post-tumor inoculation). The treatment was given three times per week. At this time point, animals were euthanized for the isolation of TdLNs and tumors, and cell suspensions were processed for antibody staining using anti-CD4, anti-CD8, anti-CD49b, anti-IFN-γ, and anti-granzyme B (GrzB). Further analysis was performed using flow cytometry, gating out cTreg and Tr1-like cells. The figure shows graphs depicting the frequencies of total CD8+ T cells and CD8+IFN-γ+ T cells in TdLN **(A)**, and total CD8+ T cells, CD8+IFN-γ+ T cells, and CD8+GrzB+ T cells in TILs **(B)**. The same analysis was performed for the CD4+ T cell compartment, in which total CD4+ T cells and CD4+IFN-γ+ T cells in TdLN **(C)** and total CD4+ T cells, CD4+IFN-γ+ T cells, and CD4+GrzB+ T cells in TILs **(D)** are shown. Data from three independent experiments are shown as the mean ± SEM. n = 3–6 mice per experiment. * p < 0.05; ** p< 0.01; *** p < 0.001; **** p< 0.0001; ns, not significant according to one-way ANOVA (multiple comparison) for panels **(A, C)** and Mann–Whitney test (for panels **B, D**). **(E)** RAG-KO mice inoculated with 10^5^ B16 melanoma cells and treated as above.

Furthermore, we included the phenotypic analysis on effector CD8+ T and CD4+ T cells. As described above for cTreg and Tr1 cells, CD8+T and CD4+ T cells remained in similar frequencies in the lymph nodes of tumor animals, treated or not with anti-CD29, ∼25% for both subsets (**Figures 6A, C**, left graphs). In TILs, CD8+ T cells also remained unchanged (∼20%), but CD4+ T cells reduced their frequencies from ∼25% in controls animals versus ∼15% in the treated group (**Figures 6B, D**, left graphs). When testing the expression of IFN-γ on these cells in TdLN, we did not find major differences, although there is a tendency on higher frequencies of both CD8+IFN-γ+ T and CD4+IFN-γ+ T cells in treated mice (**Figures 6A, C**, right graphs). This tendency was clearly present in TILs, where tumor animals treated with anti-CD29 antibody showed higher frequencies of CD8+IFN-γ+ T cells (∼20% versus ∼15% in controls) and CD8+GrzB+ T cells (∼15% versus ∼9% in controls) (**Figure 6B**, center and right plots).

These data suggest that the treatment with anti-CD29 antibody on tumor-bearing animals inhibits tumor growth by promoting T-cell-mediated immunity, as anti-CD29 administration did not exert anti-tumor effects in the absence of B and T cells (**Figure 6E**).

**Figure 7:**
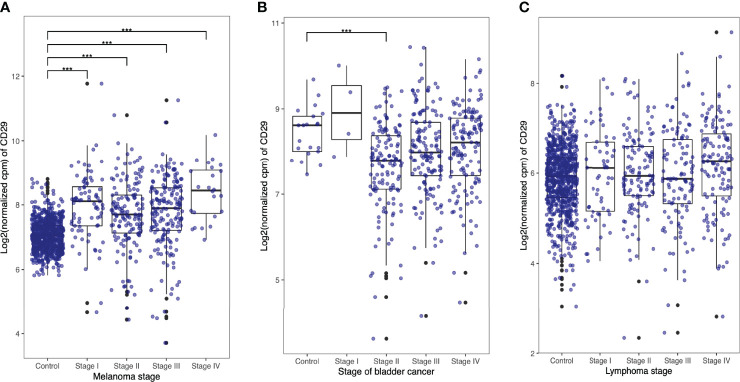
Human tumor cells express CD49b and CD29. RNA-Seq data from healthy tissues (control) was downloaded from the Genotype-Tissue Expression (GTEx) (v8) project (https://gtexportal.org). Differential gene expression was assessed using R/edgeR by applying trimmed mean of M-values (TMM) normalization. Respective cancer and normal tissue sample data were normalized together to avoid composition bias. Expression of genes CD29 (ENSG00000150093) and CD49b (ENSG00000164171) was evaluated in melanoma **(A)**, bladder **(B)**, and lymphoma **(C)** cancer samples.

Lastly, we performed CD29 gene expression analysis on human cancer samples as a reasonable approximation to the clinical setting. We used The National Cancer Institute GDC data portal and Genotype-Tissue Expression (GTEx) (v8) project database and compared CD29 expression levels on stages I, II, III, and IV melanoma, bladder, and lymphoma cancer cells samples (**Figures 7A–C**, respectively). As depicted in **Figure 7**, CD29 expression is upregulated in melanoma samples as the disease progresses (**Figure 7A**). For the case of bladder and lymphoma, CD29 expression seems to be present in some individuals, but no marked pattern or statistical difference was obtained. All together, these results suggest that CD29 could serve as a target for immunotherapy, although a detailed gene expression and protein production analysis should be carried out in different tumors (and stages) obtained from patients.

## Discussion

The role of FoxP3+Treg cells in solid tumors is fundamental. Their comprehensive study facilitated the discovery of key molecules that now are the targets for immunotherapy. On the contrary, Tr1 cells have not attracted the same interest, but reports have described their presence and function in some tumor models. In this study, we were interested in comparing cTreg and Tr1 cells in tumor-bearing animals. First, we defined cTreg cells as those CD4+ T cells expressing FoxP3, for which we used FoxP3^GFP^ reporter animals (for easing the detection of this transcription factor’s expression). Conversely, Tr1 cells were identified as CD4+ T cells negative for FoxP3 but expressing CD49b, the integrin α2 (13, 37) (**Figure 1**). The production of IL-10, one of the hallmarks of suppressive cells, was found in 10% of cTreg and Tr1 cells in naive animals (**Figure 1C**), confirming the identity of these regulatory cells, and criteria to discriminate between these cells subsets based on FoxP3 and CD49b (13). The decision of not including LAG-3 as a marker was made after the questioning of the specificity of the LAG-3 marker by White and Wraith, who reported that, while 50% of antigen-specific CD4+IL-10+ T cells obtained *ex vivo* from EAE mice were positive for CD49b, LAG-3 was found in the majority of CD4+ T cells, regardless of their IL-10 production, thus rendering it a non-specific marker (11). This same study reported that only a small proportion of *in vitro*-induced CD4+IL-10+ T cells was positive for both CD49b and LAG-3, and while almost 60% of IL-10+ T cells were CD49b+, only 10% were LAG-3+ (11). When we analyzed the presence of cTreg and Tr1 cells in tumor-bearing animals, comparing between the TdLNs and TILs at day 20 after tumor induction, we found that both populations are present in the organs in different proportions, 4% and 30%, respectively, suggesting that they are distinct cell subsets differentially receiving/responding to cues from these microenvironments. This piece of data is very important because cTreg cells have been widely recognized as the major population of cells contributing to tumor growth, leaving Tr1 cells underappreciated; our study shows that Tr1 cells are more abundant than cTreg in TILs (**Figure 2B**). This finding is relevant when we are looking for strategies to eliminate tumors. To elucidate the origin of the high proportion of Tr1 cells in the TILs fraction, we next performed kinetic experiments and evaluated the proliferative capacity of both Treg subsets. In coherence with our previous results, we found that both subsets behave differently depending on the site where they are residing. cTreg and Tr1 cells in the TdLNs become less abundant during the first days of tumor growth, suggesting that the cells may be dying or migrating out of the lymph node (**Figures 2A, B**). Interestingly, we found that Tr1 cells are present in high frequencies since early in the tumor onset process, comprising over 20% of the CD4+ population 1 week after the tumor induction, while the cTreg fraction comprised <5% of the CD4+ T cell subset at the same time point (**Figure 2B**). While Tr1 cells frequency is stable between all analyzed time points, cTreg frequency increases in the TILs fraction at later time points. Taking into consideration these results, we can speculate that the frequency of these subsets in the TILs is being mediated by different mechanisms: an active recruitment of cTreg and Tr1 cells, proliferation, or *de novo* differentiation. To determine if these subsets are actively proliferating at the tumor site, we performed a BrdU incorporation study. We found that cTreg cells are more proliferative than Tr1 cells, which have a low proliferative rate at the analyzed time points (**Figures 1C–E**). Thus, we can speculate that while cTreg cells increase their frequency in the tumor site by actively proliferating (although we cannot rule out other mechanisms), high frequency of Tr1 cells in the tumor is not due to their proliferation at the analyzed time points, and it is possible that other mechanisms such as active recruitment from the lymph nodes or *de novo* differentiation are important for mediating their presence in the tumor.

Once again, we can state that cTreg cells are different from Tr1 cells in the tumor context, with Tr1 cells being more abundant than cTreg cells in TILs, but less proliferative. Differences in survival, dynamics in recruitment or recirculation, and cellular differentiation are all possible responses to be considered in future studies.

As mentioned above, several molecules have been identified as markers of T cells with regulatory properties. Among them, we chose Nrp-1, PD-1, CD73, IFN-γ, and IL-10 to be evaluated in cTreg and Tr1 cells. In naive animals, the expression of Nrp-1 and PD-1 was higher in Tr1 cells, whereas the level of CD73 was reduced in about 50% on Tr1 cells versus cTreg cells (**Supplementary Figure 1**). In tumor-bearing animals, the expression of Nrp-1 behaves similarly as in naive draining lymph nodes on cTreg and Tr1 cells, but PD-1 and CD73 show opposite trends within the tumor versus naive (or TdLN). These results suggest that cTreg and Tr1 cells may use a preferential mechanism of suppression. It has been reported that Nrp-1 plays a role in “infecting” CD4+ T cells to differentiate into regulatory cells (38). This molecule has been found to be relevant for Treg phenotype stability in the tumor context, and its absence induces tumor clearance (39). On the other hand, both CD73 and PD-1 have been described as a potent mediator of the immunosuppressive microenvironment in the tumor (40, 41).

Furthermore, we found that IL-10 expression is higher in Tr1 cells than in cTreg cells in tumor mice, reaching the highest frequencies in TILs (**Figure 3E**). For IFN-γ, no differences were found between cTreg and Tr1 cells in the different sites, but TILs showed around 10% of IFN-γ+ cTreg and of IFN-γ+ Tr1 cells in this tissue (**Figure 3E**, right graph). While Tr1 cells cytokine signature includes significant secretion of IFN-γ, its secretion by cTreg has been related to an unstable immunosuppressive phenotype, which highlights the possible differences between the two subsets in the tumor microenvironment (39).

The discrimination between cTreg and Tr1 cells in this study is based on FoxP3 and CD49b expression, contributing to previous reports in which CD49b is postulated as a marker for Tr1 cells. With the objective of intervening with Tr1 cells in the tumor setting, we hypothesized that the administration of an antibody against CD29 should interrupt with CD49b binding. In this part of the study, animals were inoculated with tumor cells and, around day 9, received the first injection of anti-CD29 antibody (200 ng *via* i.p.). The treatment was given three times per week until day 20, and we did not observe detrimental effects on the animals (changes in behavior resulting of pain, toxicity, or other discomfort). Our striking results show that anti-CD29 antibody inhibits the growth of B16 melanoma and MB49 cells (**Figures 4A–F**). In the case of mice receiving EL-4 cells, the treatment did not have an effect (**Figures 4G–I**). We screened these three tumor cell lines for CD49b and CD29 expression, finding a correlation between the expression of these molecules and the results obtained with the administration of the anti-CD29 antibody (**Supplementary Figure 2**). In other words, the effectiveness of anti-CD29 treatment was dependent on CD49b/CD29 expression on tumor cells. Additionally, preliminary data eliminate the possibility of anti-CD29 inducing direct apoptosis and/or necrosis on tumor cells, as demonstrated in **Supplementary Figure 3**, in which no apoptosis or necrosis was found upon treatment. H&E staining of the tumors demonstrates aggressive tumor growth and infiltration in control animals, whereas those receiving anti-CD29 display tumors with leukocyte infiltration (**Figures 5A–D**). Furthermore, because it is possible that antibody binding to the surface of cancer cells may be facilitating antibody-dependent cellular cytotoxicity [or ADCC (42)], we performed the tumor growth experiment using RAG-KO immunodeficient animals, which are deprived of B- and T-cell-mediated responses (43). Our results showed no difference in tumor growth curves, indicating that the adaptive immune response is involved in anti-CD29 treatment. Overall, our study presents a characterization of Treg subsets present in tumors, leading to CD29 as putative target for immunotherapy, since its administration strikingly inhibits melanoma and MB49 tumor growth. This process is mediated by T-cell-dependent immunity, skews T-cell phenotype toward an inflammatory profile, and may require the presence of CD29 on the tumor itself. Human gene expression analysis indicates that CD29 is present in cancer cells and that its expression could vary depending on disease stage.

## Data Availability Statement

The original contributions presented in the study are included in the article/**Supplementary Material**. Further inquiries can be directed to the corresponding author.

## Ethics Statement

The animal study was reviewed and approved by Committee guidelines from the Faculty of Medicine, Universidad de los Andes, and the Agencia Nacional de Investigación y Desarrollo (ANID).

## Author Contributions

PC-K performed experiments and analyses and wrote the paper. FG-J and JD-S performed experiments and their analyses. AE, WG-A, FA-M, and RN analyzed and discussed data. KP-L conceived and designed experiments, analyzed data, and wrote the manuscript. All authors contributed to the article and approved the submitted version.

## Funding

This work was supported by National Agency for Investigation and Development (ANID) through FONDECYT Regular #1210654 (KP-L), #1190411 (FA-M), and basal funding for Scientific and Technological Center of Excellence (IMPACT, #FB210024) (FA-M and KP-L).

## Conflict of Interest

Author FA-M was employed by Cells for Cells.

The remaining authors declare that the research was conducted in the absence of any commercial or financial relationships that could be construed as a potential conflict of interest.

## Publisher’s Note

All claims expressed in this article are solely those of the authors and do not necessarily represent those of their affiliated organizations, or those of the publisher, the editors and the reviewers. Any product that may be evaluated in this article, or claim that may be made by its manufacturer, is not guaranteed or endorsed by the publisher.

## Supplementary Material

Supplementary Figure 1 Differential expression of Nrp-1, PD-1 and CD73 on cTreg versus Tr1 cells. Two x 10^5^ B16 cells were injected into the right flank of mice and tumor growth was monitored and measured with caliper every other day. Between day 18-20 post-inoculation, mice were euthanized for cell phenotyping in naïve ingüinal lymph nodes (Naïve), tumor-draining lymph nodes (TdLNs) and tumor-infiltaring lymphocytes (TILs) using flow cytometry. The level of expression of Nrp-1 **(A)**, PD-1 **(B)** and CD73 **(C)** on CD4+ T cells, cTreg cells and Tr1 cells were evaluated, and its mean fluorescence intensity (MFI) values are shown in the indicated graphs. Data from two independent experiments are shown as the mean ± SEM. n = 3-6 mice per experiment. * p < 0.05; ** p< 0.01; *** p < 0.001; ns not significant according to One-Way ANOVA (multiple comparison).

Supplementary Figure 2 Expression of CD49b and CD29 on tumor cell lines. B16 melanoma, MB49 and EL-4 tumor cell lines were cultured *in vitro*. Cells were harvested and stained with antibodies to check for the expression of CD49b and CD29 by flow cytometry. **(A)** Representative dot plots depicting the expression of CD73, CD49b and CD29. **(B)** Graphs showing the frequencies of tumor cells CD49b+ (top) or CD29+ (bottom). This experiment was performed two times.

Supplementary Figure 3 Determination of apoptosis and necrosis on tumor cells incubated with anti-CD29 antibody. Indicted tumor cells lines were cultured *in vitro* at 70% of confluency. Adherent and suspension cells were recovered and stained for 7AAD and Annexin-V. **(A)** Representative flow cytometry plots depicting 7AAD and Annexin-V staining on B16, MB49 and EL4 tumor cells. **(B–D)** Show the frequencies of live (left), apoptotic (center) and necrotic (right) B16, MB49 and EL4 tumor cells, respectively.
