# Supplementary figures and images for: CD49b Targeting Inhibits Tumor Growth and Boosts Anti-tumor Immunity
Source: Front Oncol. 2022 Jul 4;12:928498. doi: 10.3389/fonc.2022.928498 (PMC9291404; doi:10.3389/fonc.2022.928498)

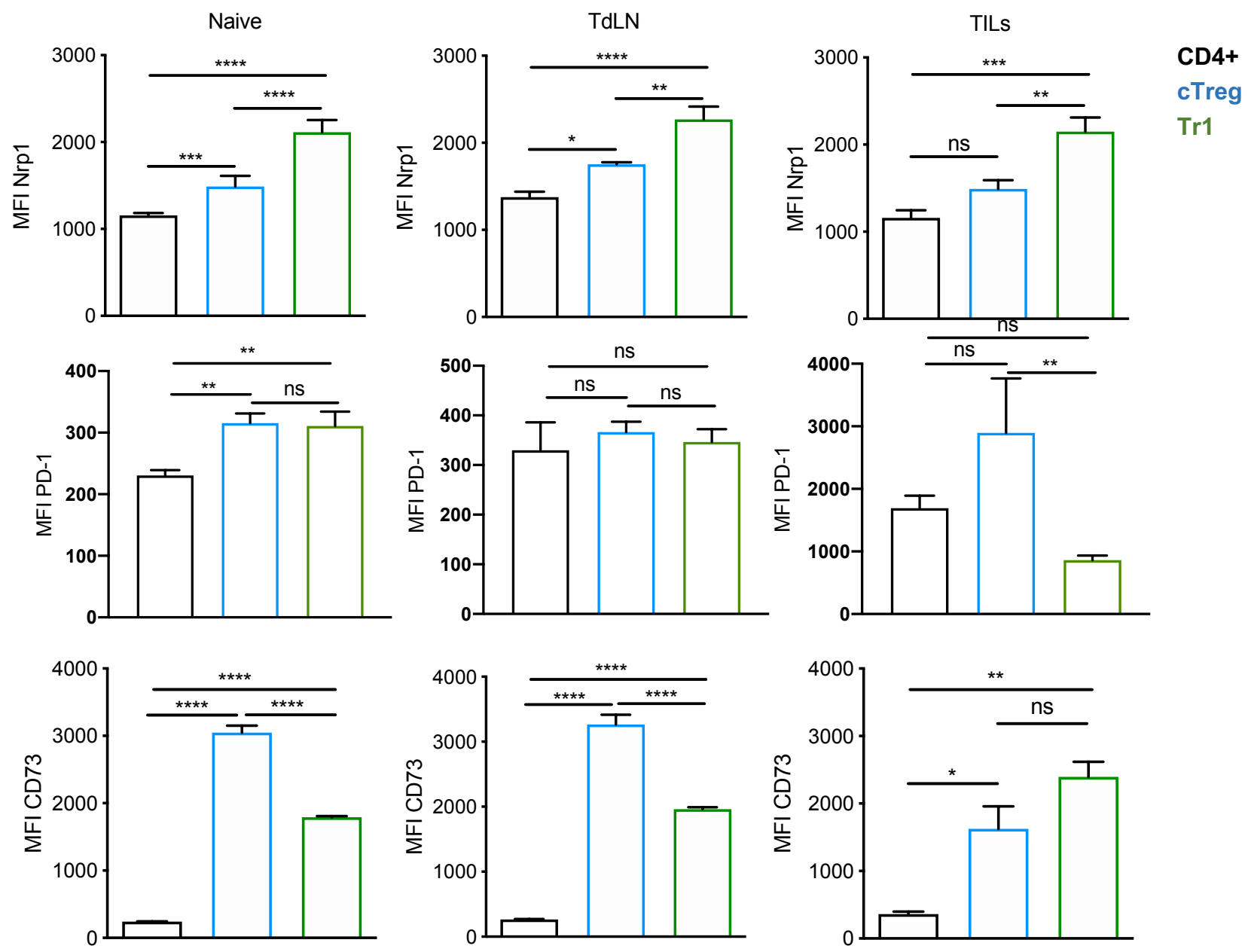

Supplement: Supplementary Figure 1 — Differential expression of Nrp-1, PD-1 and CD73 on cTreg versus Tr1 cells. Two x 105 B16 cells were injected into the right flank of mice and tumor growth was monitored and measured with caliper every other day. Between day 18-20 post-inoculation, mice were euthanized for cell phenotyping in naïve ingüinal lymph nodes (Naïve), tumor-draining lymph nodes (TdLNs) and tumor-infiltaring lymphocytes (TILs) using flow cytometry. The level of expression of Nrp-1 (A), PD-1 (B) and CD73 (C) on CD4+ T cells, cTreg cells and Tr1 cells were evaluated, and its mean fluorescence intensity (MFI) values are shown in the indicated graphs. Data from two independent experiments are shown as the mean ± SEM. n = 3-6 mice per experiment. * p < 0.05; ** p< 0.01; *** p < 0.001; ns not significant according to One-Way ANOVA (multiple comparison). [file DataSheet_1.pdf]

A

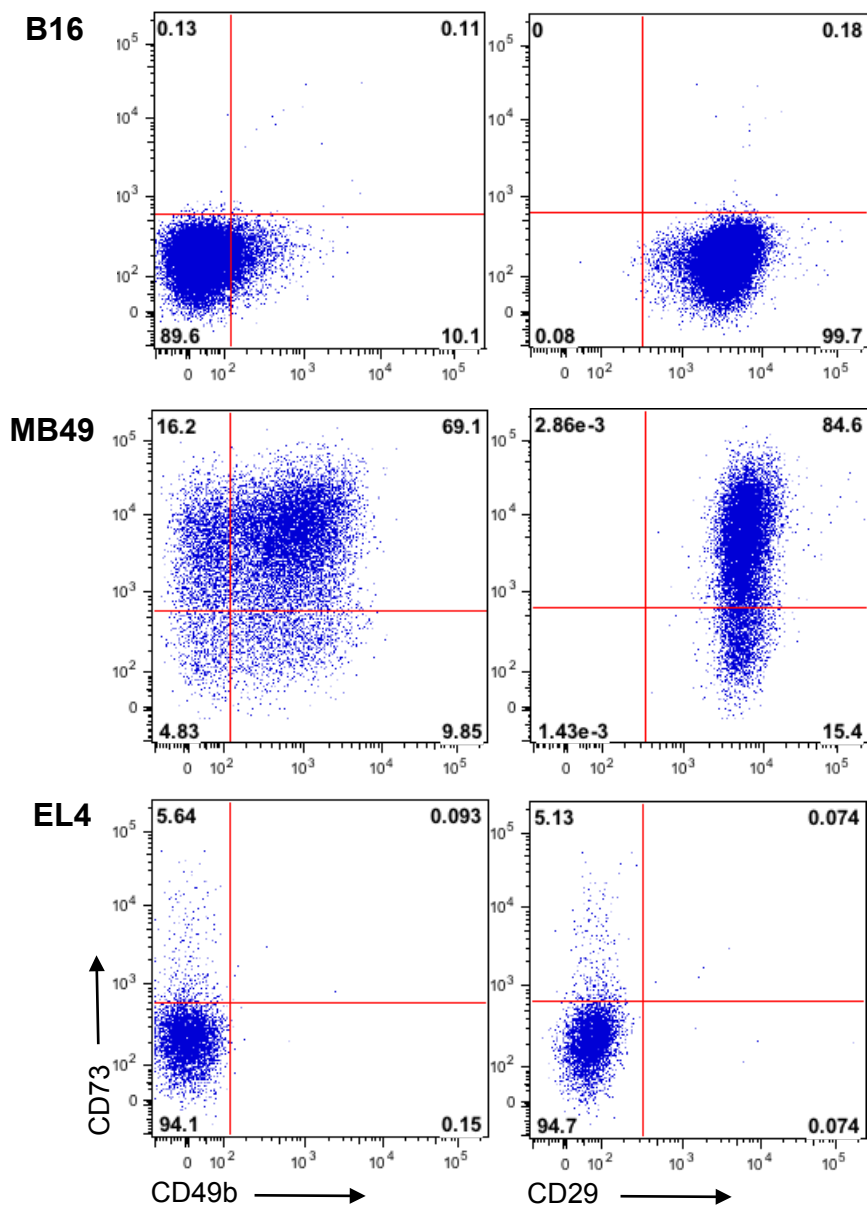

B

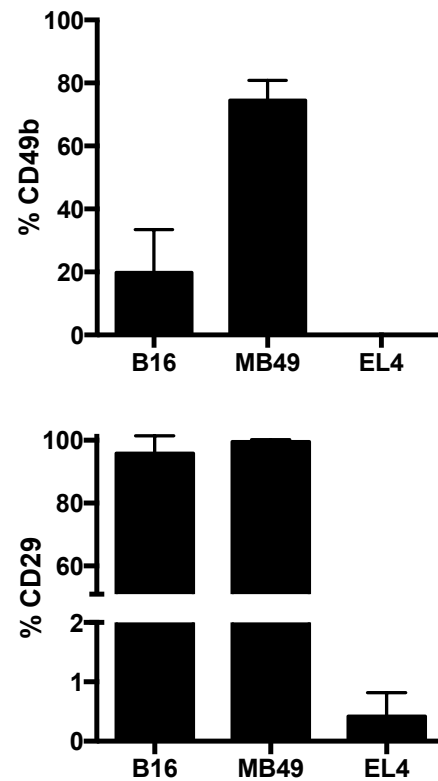

Supplement: Supplementary Figure 2 — Expression of CD49b and CD29 on tumor cell lines. B16 melanoma, MB49 and EL-4 tumor cell lines were cultured in vitro. Cells were harvested and stained with antibodies to check for the expression of CD49b and CD29 by flow cytometry. (A) Representative dot plots depicting the expression of CD73, CD49b and CD29. (B) Graphs showing the frequencies of tumor cells CD49b+ (top) or CD29+ (bottom). This experiment was performed two times. [file DataSheet_2.pdf]

A

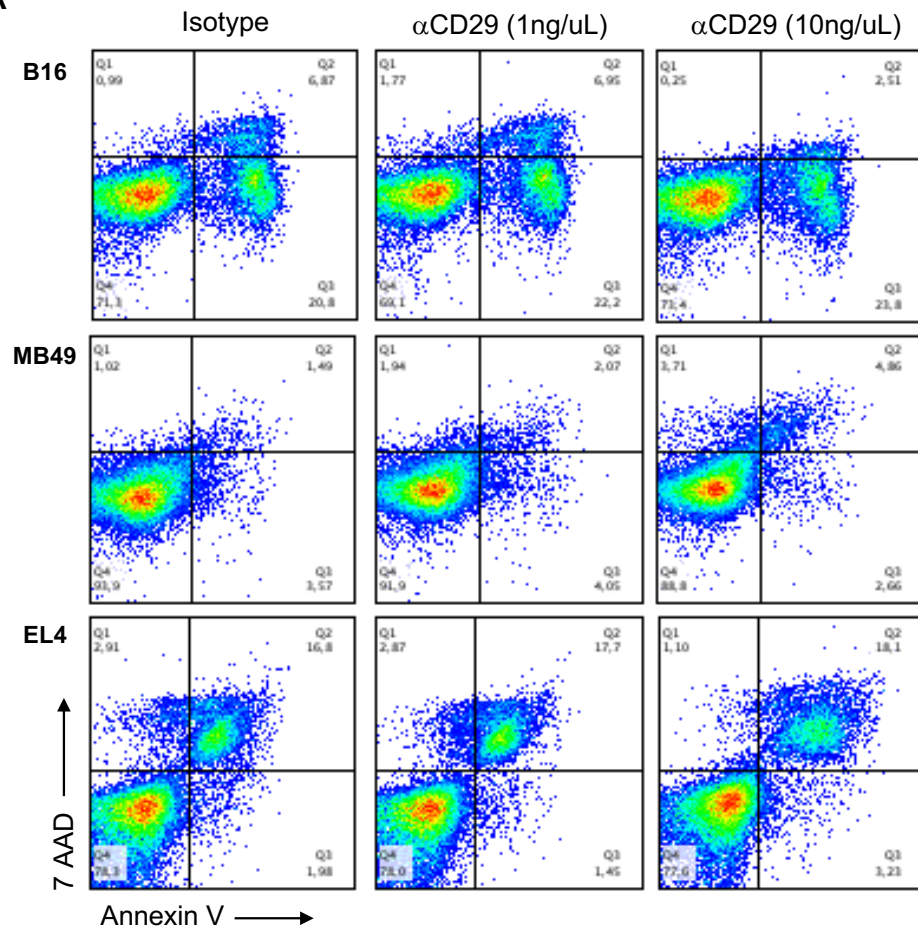

B

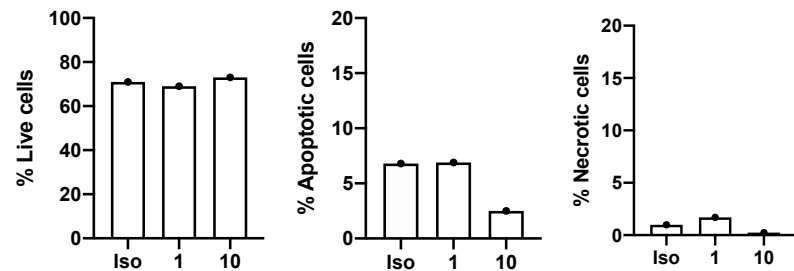

C

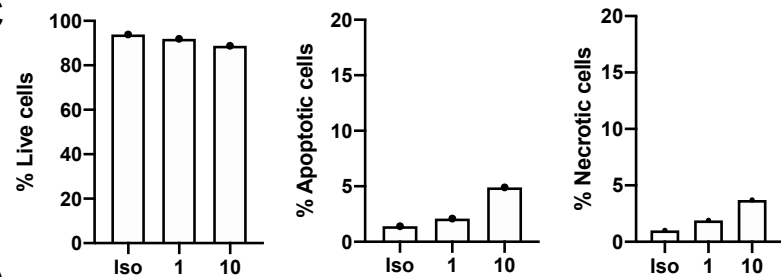

D

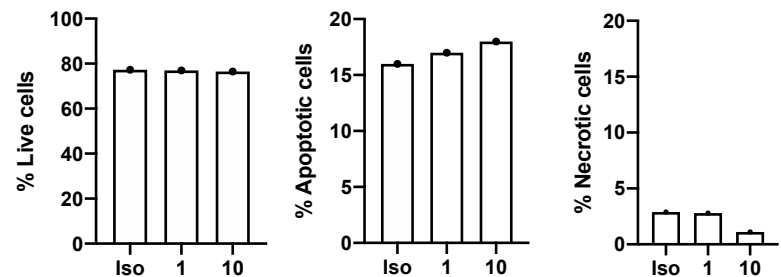

Supplement: Supplementary Figure 3 — Determination of apoptosis and necrosis on tumor cells incubated with anti-CD29 antibody. Indicted tumor cells lines were cultured in vitro at 70% of confluency. Adherent and suspension cells were recovered and stained for 7AAD and Annexin-V. (A) Representative flow cytometry plots depicting 7AAD and Annexin-V staining on B16, MB49 and EL4 tumor cells. (B–D) Show the frequencies of live (left), apoptotic (center) and necrotic (right) B16, MB49 and EL4 tumor cells, respectively. [file DataSheet_3.pdf]
